# [Retracted] Metformin mitigates PLCε gene expression and modulates the Notch1/Hes and androgen receptor signaling pathways in castration-resistant prostate cancer xenograft models

**DOI:** 10.3892/ol.2025.15082

**Published:** 2025-05-12

**Authors:** Qi Li, Ke Xu, Jianguo Tian, Zhicheng Lu, Jianming Pu

Oncol Lett 22: 715, 2021; DOI: 10.3892/ol.2021.12976

Following the publication of this paper, it was drawn to the Editor's attention by a concerned reader that certain of the histological data shown in Fig. 2D-F on p. 4, and the AR expression data shown in Fig. 5B on p. 6 had already appeared in a previously published article in the journal *European Urology* written by different authors at different research institutes. Owing to the fact that the contentious data in the above article had already been published prior to its submission to *Oncology Letters*, the Editor has decided that this paper should be retracted from the Journal. The authors were asked for an explanation to account for these concerns, but the Editorial Office did not receive a reply. The Editor apologizes to the readership for any inconvenience caused.

